# Diaphorin, a polyketide synthesized by an intracellular symbiont of the Asian citrus psyllid, is potentially harmful for biological control agents

**DOI:** 10.1371/journal.pone.0216319

**Published:** 2019-05-02

**Authors:** Tomoko Yamada, Masato Hamada, Paul Floreancig, Atsushi Nakabachi

**Affiliations:** 1 Department of Environmental and Life Sciences, Toyohashi University of Technology, Toyohashi, Aichi, Japan; 2 Department of Chemistry, University of Pittsburgh, Pittsburgh, Pennsylvania, United States of America; 3 Electronics-Inspired Interdisciplinary Research Institute (EIIRIS), Toyohashi University of Technology, Toyohashi, Aichi, Japan; US Department of Agriculture, UNITED STATES

## Abstract

The Asian citrus psyllid *Diaphorina citri* Kuwayama (Hemiptera: Sternorrhyncha: Psylloidea: Liviidae) is an important pest of citrus species worldwide because it transmits *Candidatus* Liberibacter spp. (*Alphaproteobacteria*), the causative agents of an incurable citrus disease known as huanglongbing or greening disease. *Diaphorina citri* possesses a vertically-transmitted intracellular symbiont, *Candidatus* Profftella armatura (*Betaproteobacteria*), which produces diaphorin, a polyketide that is significantly toxic to mammalian cells. Diaphorin is an analog of pederin, a defensive polyketide in the body fluid of *Paederus* rove beetles (Coleoptera: Staphylinidae) that deters predators. In the present study, as a first step to assess the possibility that diaphorin is toxic to biological control agents, we assayed diaphorin activities against insects and fungi. The target cells and organisms were (a) the Sf9 cell line derived from the fall armyworm moth *Spodoptera frugiperda* (Lepidoptera: Noctuidae), (b) the pea aphid *Acyrthosiphon pisum* (Hemiptera: Sternorrhyncha: Aphidoidea: Aphididae), a phloem sap-sucking insect that is closely related to psyllids, (c) the Asian lady beetle *Harmonia axyridis* (Coleoptera: Coccinellidae), one of the major predators of *D*. *citri*, and (d) the budding yeast *Saccharomyces cerevisiae* (Ascomycota: Saccharomycetes) as a model of fungal pathogens. For a comparison, we also evaluated pederin activities. The results of our analyses revealed the following: (1) Diaphorin and pederin are significantly toxic to the tested insects and yeast; (2) Their toxicities vary widely among the target cells and organisms; (3) Diaphorin is generally less toxic than pederin; (4) The toxicities of diaphorin and pederin are considerably different in the Sf9 insect cell line and *S*. *cerevisiae*, but similar in *A*. *pisum* and *H*. *axyridis*; and (5) The amount of diaphorin contained in *D*. *citri* is toxic to all of the tested cells and organisms, suggesting that this polyketide is potentially harmful for biological control agents.

## Introduction

The Asian citrus psyllid *Diaphorina citri* Kuwayama (Hemiptera: Sternorrhyncha: Psylloidea: Liviidae) is a serious pest of citrus trees worldwide because it transmits *Candidatus* Liberibacter spp. (*Alphaproteobacteria*), the causative agents of a devastating citrus disease known as huanglongbing (HLB) or greening disease [1]. All commercial citrus cultivars are susceptible to HLB, and a long latent period after infection facilitates the rapid spread of the disease, which is a severe threat to the citrus industry. Because HLB is currently incurable, controlling the *D*. *citri* vector is the most crucial aspect of HLB management. The application of chemical insecticides is presently the primary option for controlling *D*. *citri*. However, a more sustainable strategy is warranted, including biological control with natural enemies [1–8], partly because of the global increase in the resistance of *D*. *citri* to various pesticides [9–12].

**Fig 1:**
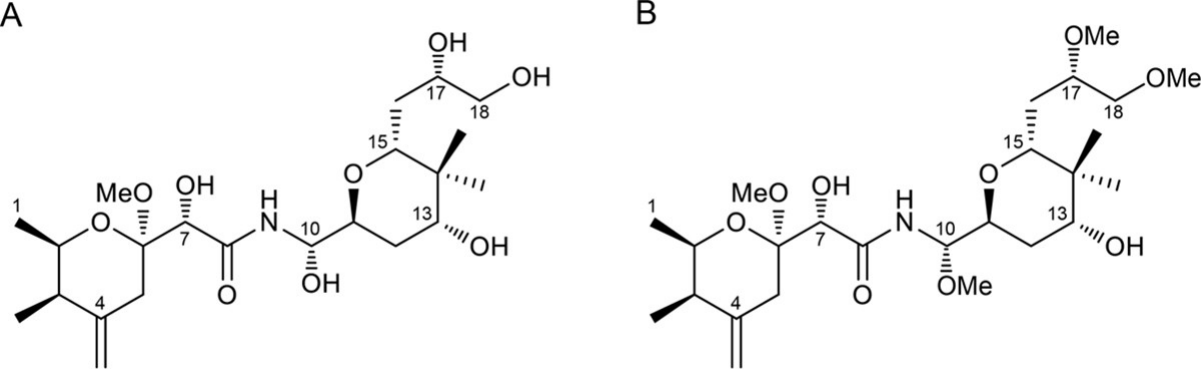
**Structures of diaphorin (A) and pederin (B).** Diaphorin has hydroxyl groups at C10, C17, and C18, whereas these groups are methylated in pederin.

The *D*. *citri* hemocoel contains a symbiotic organ called the bacteriome, which harbors two distinct intracellular symbionts, namely *Ca*. Carsonella ruddii (*Gammaproteobacteria*) and *Ca*. Profftella armatura (*Betaproteobacteria*) [13–17] *Carsonella* is a typical nutritional symbiont, providing its host with essential amino acids that are scarce in the phloem sap diet [13,15,18]. In contrast, *Profftella* appears to be an organelle-like defensive symbiont, producing toxins that protect the host from natural enemies. *Profftella* has a very small genome comprising 460 kb, a large part of which is devoted to a gene set for the synthesis of a polyketide, diaphorin (Fig 1A) [13]. Diaphorin is an analog of pederin (Fig 1B), which is a defensive polyketide that accumulates in the body fluid of *Paederus* rove beetles (Coleoptera: Staphylinidae) to deter predators [19–21]. Diaphorin is significantly cytotoxic to mammalian cells, suggesting it helps protect *D*. *citri* from vertebrate predators [13]. In addition to vertebrates, *D*. *citri* has natural enemies from various lineages, including arthropod predators (e.g., lady beetles, lacewings, and spiders) [3,4,7], hymenopteran parasitoids [2,8], and entomopathogenic fungi [5,6]. As these arthropods and fungi are potentially useful biological pesticides, information regarding their susceptibility to diaphorin is essential for the successful biological control of *D*. *citri*.

In the present study, we assessed the biological activities of diaphorin against insects and fungi. Regarding insects, we used (1) the Sf9 cell line that is commonly used in insect cell cultures for recombinant protein production [22], (2) the pea aphid *Acyrthosiphon pisum* (Hemiptera: Sternorrhyncha: Aphidoidea: Aphididae), which is a phloem sap-sucking insect that is closely related to psyllids [23], and (3) the Asian lady beetle *Harmonia axyridis* (Coleoptera: Coccinellidae), which is one of the major predators of *D*. *citri* [3,4]. As a model of fungal pathogens, the budding yeast *Saccharomyces cerevisiae* (Ascomycota: Saccharomycetes) [24] was also analyzed. For a comparison, we also evaluated the activities of pederin, which is an analog of diaphorin.

## Materials and methods

### Insects

An established *D*. *citri* colony, originally collected from Amami Oshima Island, Kagoshima, Japan, was maintained on *Murraya paniculata* (Rutaceae) at 28°C with a 16-h light:8-h dark photoperiod. Strain ISO, an established parthenogenetic clone of the pea aphid *A*. *pisum*, was maintained on *Vicia faba* (Fabaceae) at 20°C with a 16-h light:8-h dark photoperiod [25]. Laboratory stocks of the multicolored Asian lady beetle *H*. *axyridis*, originally collected in Aichi, Japan, were reared at 25°C with a 16-h light:8-h dark photoperiod. The beetles were maintained on an artificial diet of lyophilized drone pupa powder (Agrisect), sucrose, and ethyl-4-hydorxybenzoate as a preservative in a weight ratio of 30:10:1 [26]. Before collecting eggs, adult *H*. *axyridis* beetles were fed on *A*. *pisum* to promote fecundity.

### Insect cell line

The Sf9 cell line derived from the pupal ovarian tissue of the fall armyworm moth *Spodoptera frugiperda* (Lepidoptera: Noctuidae) [22] was purchased from Thermo Fisher Scientific (Waltham, Massachusetts, U.S.A.).

### Preparation of diaphorin

Diaphorin was extracted and purified as previously described [13], with some modifications. Briefly, adult *D*. *citri* specimens were treated twice with methanol, and the extracts were combined and concentrated *in vacuo*. The residue was resuspended in methanol and purified in a Shimadzu (Kyoto, Japan) LC10 high-performance liquid chromatography (HPLC) system with an Inertsil ODS-3 C18 reversed-phase preparative column [5 μm, 7.6 × 150 mm, GL Science (Tokyo, Japan)] heated to 35°C. The mobile phase was isocratic 20% acetonitrile in water, with a flow rate of 1.5 mL/min. Diaphorin was detected at a wavelength of 200 nm. The purified samples were combined and dried *in vacuo*. Diaphorin was re-dissolved in methanol and quantified in an HPLC system as described above, except the mobile phase was 15% acetonitrile in water, with a flow rate of 1.0 mL/min, and an Inertsil ODS-3 analytical column (5 μm, 4.0 × 250 mm, GL Science) was used. Known amounts of synthesized pederin (see below) were used as standards. The purified diaphorin was stored at −20°C until used.

### Preparation of pederin

Pederin was synthesized as previously described [27], using the nitrile group as a precursor to the *N*-acyl aminal, which allowed the synthesis from commercially available materials to be completed in 10 steps. Dried samples were stored at −20°C until used.

### Evaluation of the biological activities of diaphorin and pederin

#### Sf9 cells

Frozen cells were thawed and cultured in Sf-900 III SFM medium (Thermo Fisher Scientific) containing 25 U/mL penicillin and 25 μg/mL streptomycin. The cells were cultured at 27°C with shaking (125 rpm on an orbital shaker). Various concentrations (200 nM–20 mM) of diaphorin and pederin were prepared in 50% (v/v) methanol/water, of which 10 μL was added to 1990 μL of Sf-900 III SFM medium, resulting in media containing diaphorin or pederin at a final concentration of 1 nM–100 μM. After four successive cultivations in normal Sf-900 III SFM medium, live Sf9 cells were inoculated to the polyketide-containing media at a final cell density of 5.0 × 10^5^ cells/mL, and cultured as described above. Control cells were cultured in media containing only 0.5% volume of 50% (v/v) methanol/water (solvent of the polyketides). After 48 h cultivation, the number and proportion of live and dead cells were determined with the Tali Viability Kit—Dead Cell Red and the Tali Image-Based Cytometer (Thermo Fisher Scientific) according to the manufacturer’s instructions. All experiments were repeated five times.

#### Aphids

To securely administer known amounts of polyketides into the insect body, we used the injection method for compound delivery. Twelve-day-old parthenogenetic adult *A*. *pisum* females were individually weighed on an electronic balance and their volumes were calculated assuming a specific gravity of 1.0. Additionally, 100 μM solutions of diaphorin or pederin dissolved in 10% (v/v) methanol/water were prepared. Using thin glass capillaries connected to the CellTram vario microinjector (Eppendorf), solutions corresponding to 5% of the volume of each individual were injected into the hemocoel of aphids to achieve final polyketide concentrations of 5 μM within the aphid body. Control aphids were injected with the same amount of 10% (v/v) methanol/water alone. After injection, each aphid was transferred onto a seedling of *V*. *faba* and reared individually in a separate cage kept at 20°C with a 16-h light:8-h dark photoperiod. Aphid survival was checked every 24 h for 7 days. For Kaplan–Meier analysis, the event (death = 1) was recorded per each individual. Three independent experiments (five individuals per treatment in each experiment) were performed, giving a total of 15 cases per treatment with diaphorin or pederin.

#### Lady beetles

Diaphorin and pederin were administered to *H*. *axyridis* using the injection method. The polyketides had limited availability, so second instar larvae with a smaller body size and softer exoskeleton than adults were used for secure delivery. Insects were individually weighed and their volumes were calculated as described above. Various concentrations (100 μM–100 mM) of diaphorin and pederin dissolved in 10% (v/v) methanol/water were prepared. Solutions corresponding to 5% of the volume of each individual were injected into the hemocoel of the larvae to achieve final polyketide concentrations of 5 μM–5 mM within the body. Control insects were injected with the same amount of 10% (v/v) methanol/water alone. After injection, each insect was transferred into a separate plastic cage containing an artificial diet and water, and reared individually at 25°C with a 16-h light:8-h dark photoperiod. The survival of insects was checked every 24 h for 10 days. For Kaplan–Meier analysis, the event (death = 1) was recorded per each individual. Two independent experiments (five individuals per treatment in each experiment) were performed, giving a total number of 10 cases per treatment with diaphorin or pederin).

#### Budding yeast

*Saccharomyces cerevisiae* BY4741 cells were precultured in YPD medium containing 100 μg/mL ampicillin for 16 h at 30°C with reciprocal shaking (180 rpm). Growth was monitored by measuring the optical density of cultures at 600 nm (OD_600_) with the NanoDrop 2000c spectrophotometer (Thermo Fisher Scientific), with a 1-mm path length. Various concentrations (200 μM–200 mM) of diaphorin and pederin were prepared in 10% (v/v) methanol/water, of which 10 μL was added to 1990 μL of YPD medium, resulting in media containing diaphorin or pederin at a final concentration of 1 μM–1 mM. Yeast cells were inoculated to the polyketide-containing media, adjusting the cell density to OD_600_ = 0.01, and cultured for 48 h as before except the medium contained various concentrations of polyketides. Control cells were cultured in media containing only 0.5% volume of 10% (v/v) methanol/water (polyketide solvent). After 48 h cultivation, the cell density of each culture was analyzed by measuring the OD_600_ as described above. All experiments were repeated five times.

#### Statistical analysis

Data were analyzed with R software (version 3.4.2) [28]. Dose-response analyses of Sf9 and yeast cells were performed with the add-on package *drc* (version 3.0.1) [29] for R. The normal distribution of data was assessed with the Kolmogorov–Smirnov test [30] and the Shapiro–Wilk test [31]. Dose-response curves were estimated with log-logistic models, with 4, 3, and 2 parameters (LL.4, LL.3, and LL.2). The best-fitting model with the lowest overall standard error was selected and used to calculate the half maximal effective dose (ED_50_). Survival distributions of aphids and lady beetles were analyzed using the log-rank test and the Holm–Sidak test for multiple comparisons, when applicable [32].

## Results

### Biotoxicity of diaphorin and pederin

#### Sf9 cells

**Fig 2:**
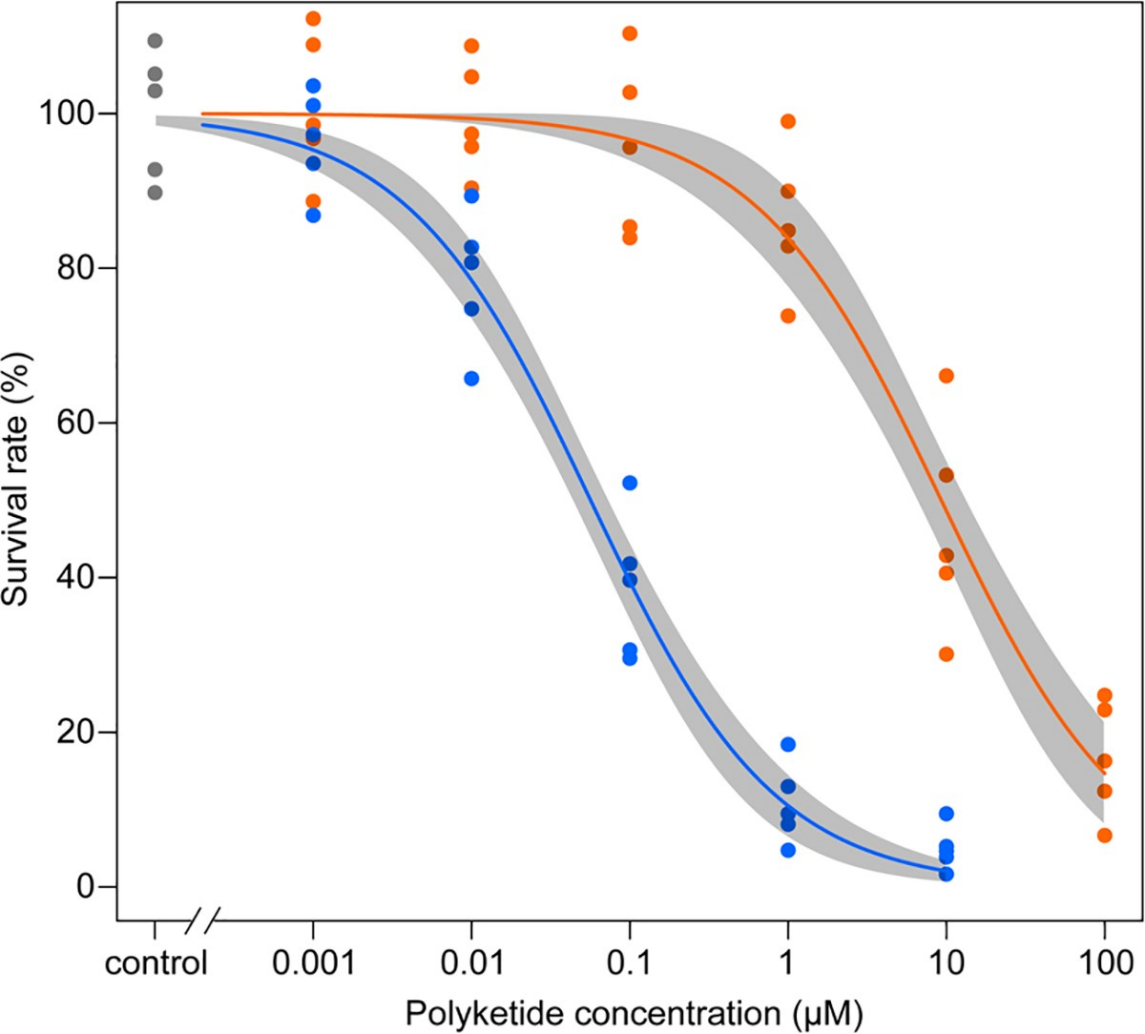
Effects of a 48-h exposure of Sf9 cells to diaphorin or pederin. Dose-response curves relating polyketide concentrations (x-axis) to Sf9 cell survival rates (y-axis). All data points (diaphorin: orange; pederin: blue; control: grey) of five repeated experiments are presented together with the lines corresponding to the fitted two-parameter log-logistic model analyzed with the statistical computing software R and its add-on package *drc*. Shaded bands represent 95% confidence intervals of the model.

Sf9 cells were cultured in medium containing 1 nM, 10 nM, 100 nM, 1 μM, 10 μM, or 100 μM diaphorin or 1 nM, 10 nM, 100 nM, 1μM, or 10 μM pederin. After a 48-h cultivation, the survival rates of Sf9 cells (number of live cells after a 48-h treatment/number of live cells at time zero) in each treatment group were calculated relative to the survival rate of the control which was not treated with polyketides. All experiments were repeated five times. The survival rates of the Sf9 cells treated with 1 nM, 10 nM, 100 nM, 1μM, or 10 μM polyketides, all five repeats of which are plotted in Fig 2, underwent a two-way analysis of variance (ANOVA). This revealed significant dosage effects (*F*_5, 59_ = 4.50, *p* < 0.001) and a significant difference in the effects of diaphorin and pederin (*F*_1, 59_ = 1.65, *p* < 0.001). To estimate dose-response curves and 50% effective doses (ED_50_), a non-linear regression analysis was performed using the log-logistic models with 4, 3, and 2 parameters [29] (Fig 2). The best-fitting model was the two-parameter logistic model that is represented by the following function:

$$f(x)=\frac{1}{1+exp(b(\log\left(x\right)-\log\left(e\right)))}$$ (1)

The coefficient *b* denotes the steepness of the dose-response curve, whereas *e* is the ED_50_. In the present case, *x* represents the polyketide dose, and the response is the Sf9 cell survival rate. The ED_50_ of diaphorin and pederin was estimated as 9.28 ± 1.65 μM and 56.5 ± 7.4 nM, respectively (Table 1), indicating that diaphorin is two orders of magnitude less toxic to Sf9 cells than pederin.

**Table 1:** Coefficients of the model fitted to the polyketide dose–Sf9 cell survival curves.

Polyketide	*b* ± SE	*e* (ED_50_) ± SE
diaphorin	0.740 ± 0.092	9.28 ± 1.65
pederin	0.746 ± 0.066	0.0565 ± 0.0074

#### Aphids

**Fig 3:**
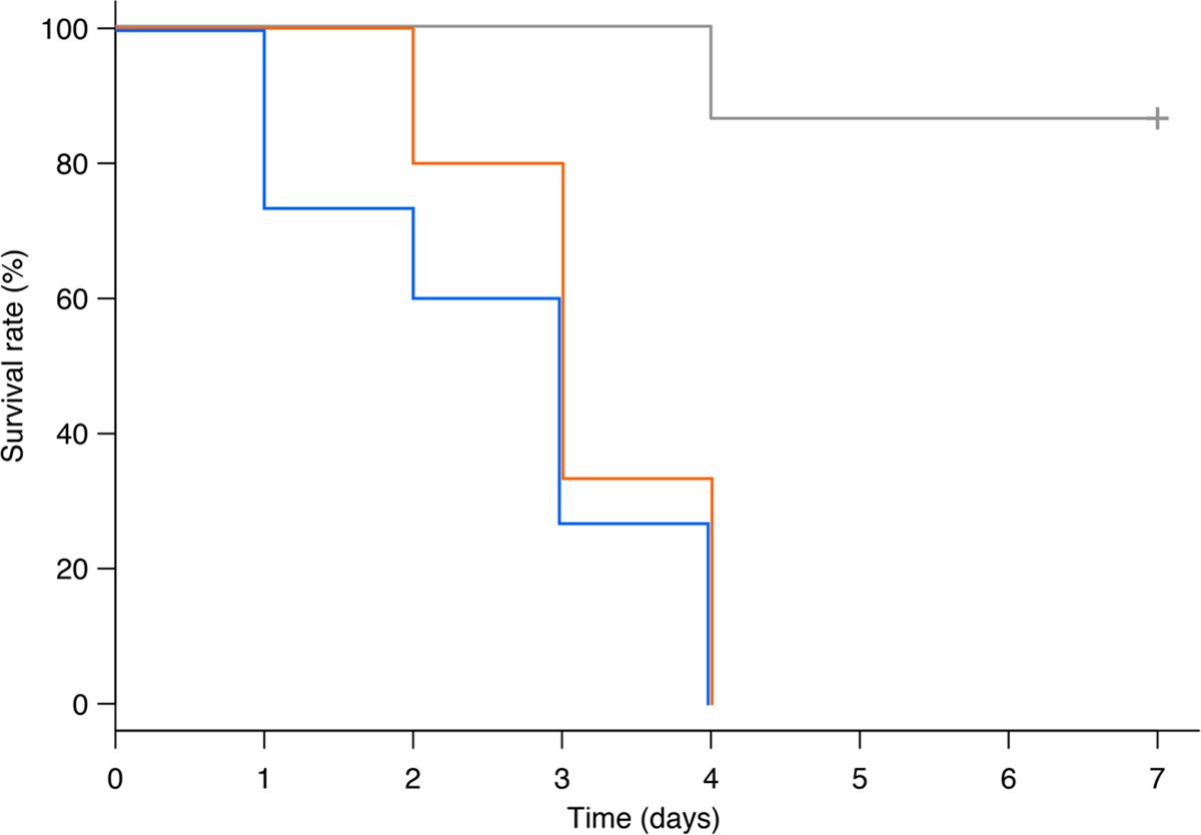
Survival rates of aphids treated with diaphorin or pederin. Kaplan–Meier survival curves of aphids treated with 5 μM diaphorin or pederin (diaphorin: orange; pederin: blue; control: grey). Data derived from three independent experiments (total of 15 individuals per treatment) were pooled and plotted on a single graph. The vertical tick mark indicates the censored time. The log-rank test and the Holm–Sidak test confirmed there were significant differences between the control and diaphorin treatment (*p* < 0.001) and between the control and pederin treatment (*p* < 0.001).

Twelve-day-old parthenogenetic adult *A*. *pisum* females were injected with diaphorin or pederin dissolved in 10% (v/v) methanol/water at a final concentration of 5 μM within the aphid body. Control aphids were injected with 10% (v/v) methanol/water alone, resulting in methanol at a final concentration of 0.5% in the insect body. Three independent experiments (five individuals per treatment in each experiment) were performed, resulting in a total of 15 cases per treatment with diaphorin or pederin. Kaplan–Meier survival curves (Fig 3) of pooled data of three independent experiments were analyzed with the log-rank test and the Holm–Sidak test with R, revealing significant differences between the control and the diaphorin treatment (*p* < 0.001) and between the control and the pederin treatment (*p* < 0.001). However, no significant differences in survival were detected between the diaphorin and pederin treatments (*p* = 0.33), indicating that they are similarly toxic to *A*. *pisum*.

#### Lady beetles

**Fig 4:**
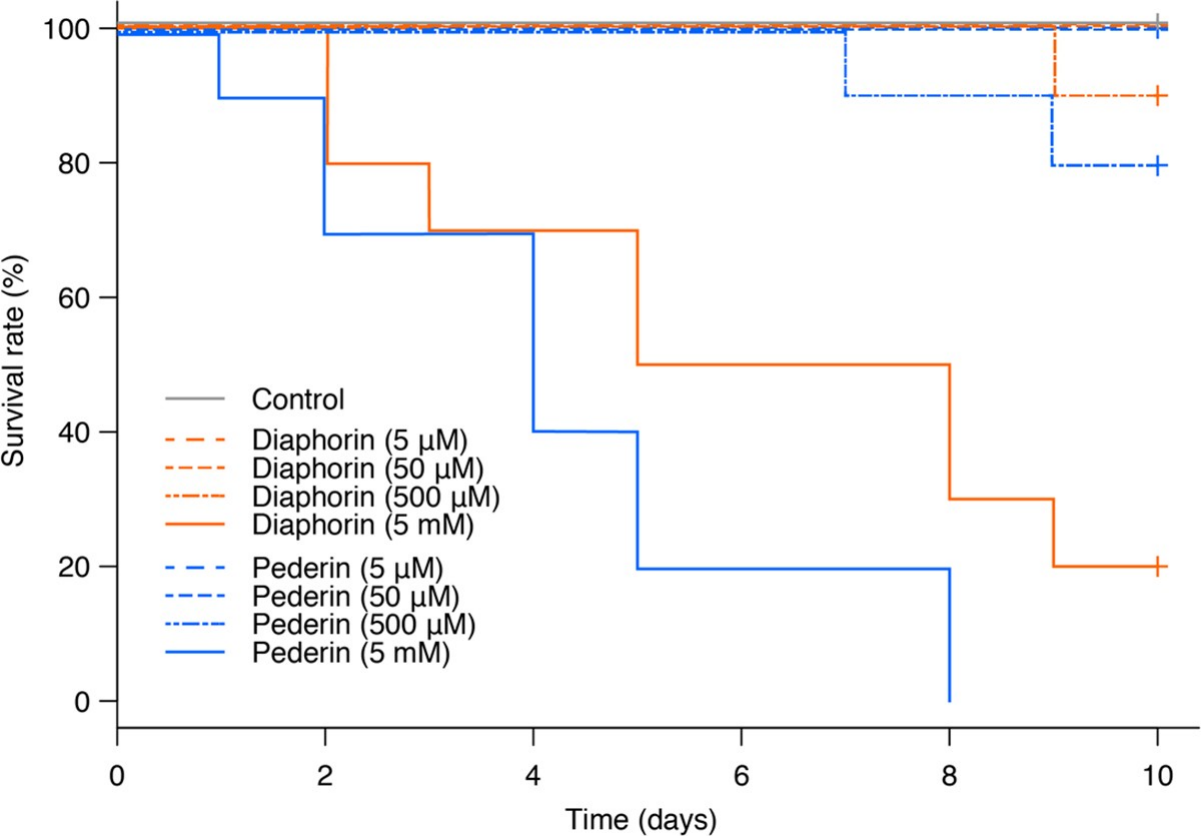
Survival rates of lady beetles treated with diaphorin or pederin. Kaplan–Meier survival curves of *H*. *axyridis* treated with 5 μM–5 mM diaphorin or pederin (diaphorin: orange; pederin: blue; control: grey). Data derived from two independent experiments (total of 10 cases per treatment) were pooled and plotted on a single graph. Vertical tick marks indicate the censored times. The log-rank test and the Holm–Sidak test detected significant differences between the control and 5 mM diaphorin treatment (*p* = 0.01315) and between the control and 5 mM pederin treatment (*p* = 0.00029).

Second instar *H*. *axyridis* larvae (weight: 1.974 ± 0.014 mg, n = 90) were injected with diaphorin or pederin dissolved in 10% (v/v) methanol/water at a final concentration of 5 μM–5 mM within the body. Control insects were injected with 10% (v/v) methanol/water alone, resulting in methanol at a final concentration of 0.5% in the insect body. Two independent experiments (five individuals per treatment) were performed, resulting in a total of 10 cases per treatment with particular concentrations of diaphorin or pederin. The Kaplan–Meier survival curves (Fig 4) of pooled data of two independent experiments were analyzed with the log-rank test and the Holm–Sidak test with R, revealing significant differences between the control and 5 mM diaphorin treatment (*p* = 0.01315) and between the control and 5 mM pederin treatment (*p* = 0.00029). However, there were no significant differences in the survival rates for the control and 5 μM–500 μM diaphorin or pederin treatments (*p* > 0.05). Additionally, there were no significant differences between 5 mM diaphorin treatment (median survival time: 6.5 days) and 5 mM pederin treatment (median survival time: 4.0 days) (*p* > 0.05), indicating they are similarly toxic to *H*. *axyridis*. Moreover, diaphorin and pederin were three orders of magnitude less toxic to *H*. *axyridis* than to *A*. *pisum*.

#### Budding yeast

**Fig 5:**
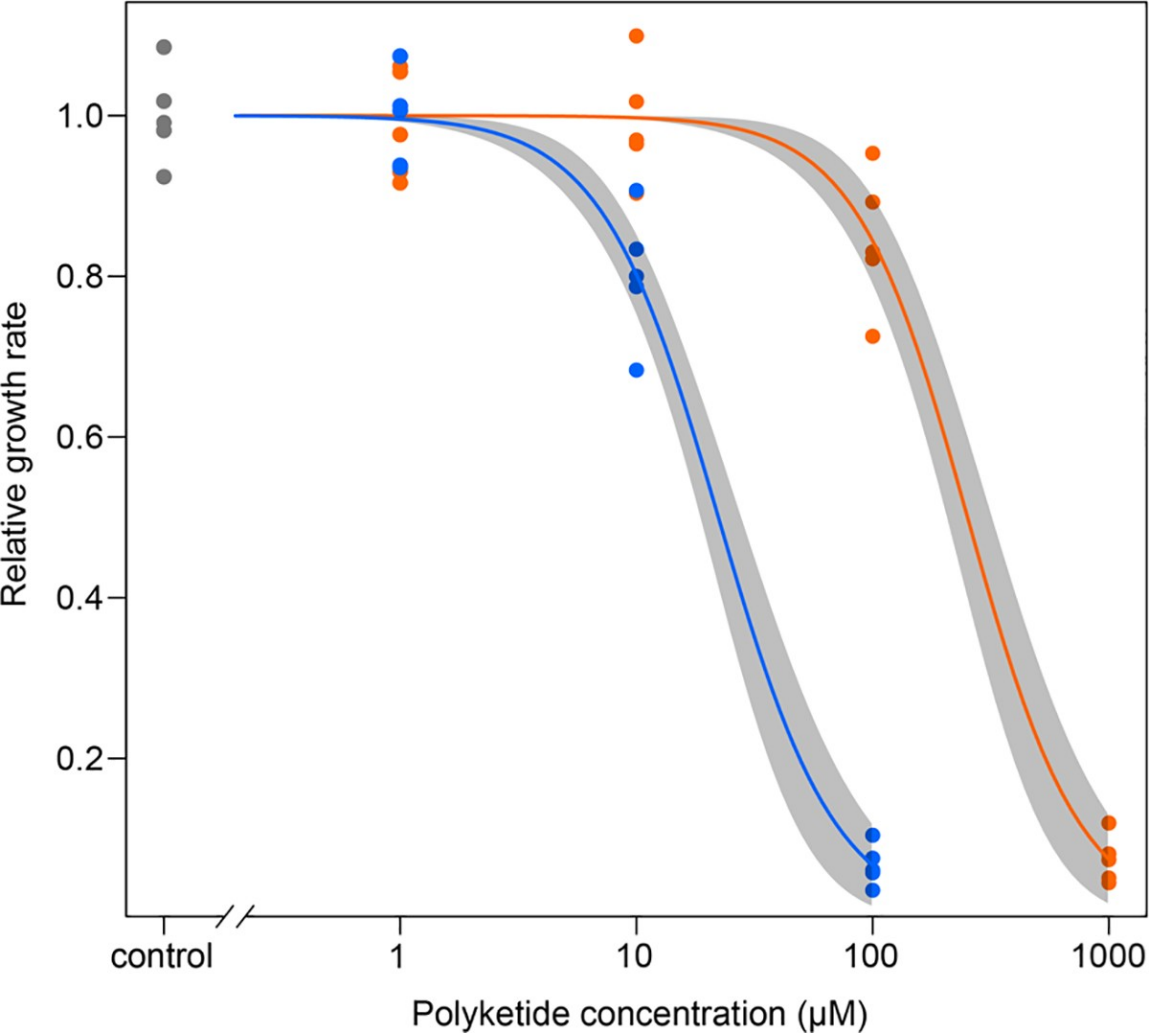
Effects of a 48-h exposure of *S*. *cerevisiae* cells to diaphorin or pederin. Dose-response curves relating polyketide concentrations (x-axis) to the relative growth rates of *S*. *cerevisiae* (y-axis). All data points (diaphorin: orange; pederin: blue; control: grey) of five repeated experiments are presented together with the lines corresponding to the fitted two-parameter log-logistic model analyzed with the statistical computing software R and its add-on package drc. Shaded bands represent 95% confidence intervals of the model.

*Saccharomyces cerevisiae* BY4741 cells were cultivated in medium containing 1 μM, 10 μM, 100 μM, or 1 mM diaphorin or 1 μM, 10 μM, or 100 μM pederin. After a 48-h cultivation, the growth rates [(OD_600_ after the 48-h treatment − OD_600_ at time zero)/OD_600_ at time zero] of the BY4741 cells in each treatment group were calculated relative to those of the control, which was not treated with polyketides. All experiments were repeated five times. The relative growth rates of BY4741 cells treated with 1 μM, 10 μM, or 100 μM polyketides, all five repeats of which are plotted in Fig 5, underwent a two-way ANOVA. This revealed significant dosage effects (*F*_3, 39_ = 1.99, *p* < 0.001) and significant differences in the effects of diaphorin and pederin (*F*_1, 39_ = 0.577, *p* < 0.001). To estimate the dose-response curves and ED_50_, a non-linear regression analysis was performed using the log-logistic models with 4, 3, and 2 parameters [29] (Fig 5). The best-fitting model was, again, the two-parameter logistic model. The ED_50_ of diaphorin and pederin was estimated as 252 ± 28 μM and 22.2 ± 2.2 μM, respectively (Table 2). These results indicated that diaphorin is about 10 times less toxic to *S*. *cerevisiae* BY4741 cells than pederin.

**Table 2:** Coefficients of the model fitted to the polyketide dose–BY4741 growth response curves.

Polyketide	*b* ± SE	*e* (ED_50_) ± SE
diaphorin	1.83 ± 0.20	252 ± 28
pederin	1.75 ± 0.19	22.2 ± 2.2

## Discussion

The present study revealed the following:

1. Diaphorin and pederin are significantly toxic to insects and fungi.
2. Their toxicities vary widely among the target cells and organisms.
3. Diaphorin is generally less toxic than pederin.
4. The toxicities of diaphorin and pederin are considerably different in Sf9 and *S*. *cerevisiae* cells, but similar in *A*. *pisum* and *H*. *axyridis*.

Diaphorin was most effective against Sf9 cells (Fig 2) and *A*. *pisum* (Fig 3), where micromolar concentrations were toxic. These concentrations were similar to the diaphorin concentrations reported to be toxic to mammalian cells [13]. Considering that *S*. *frugiperda* and *A*. *pisum* are not expected to encounter diaphorin under natural conditions, this high sensitivity may be normal for eukaryotes. Additionally, *A*. *pisum* was equally sensitive to pederin (Fig 3), but Sf9 cells were much more vulnerable to pederin, which was toxic at nanomolar concentrations (Fig 2). This discrepancy in the sensitivity to diaphorin and pederin, which was also observed for *S*. *cerevisiae* cells (Fig 5), is reminiscent of the results of a previous study that proved that mammalian cells are more susceptible to pederin (ED_50_: ~1 nM) [33] than to diaphorin (ED_50_: ~1 μM) [13].

The toxicity of pederin to eukaryotic cells is mainly attributed to its ability to bind to eukaryotic ribosomes and inhibit protein synthesis. Moreover, the C10 methoxy group of pederin (Fig 1B) is postulated to be important for ribosome binding through its hydrogen bonding and effects on conformation [33]. Diaphorin is a tri-*O*-desmethyl analog of pederin (Fig 1A), and the C10 methoxy group of pederin is replaced by a hydroxyl group in diaphorin [13,34]. While the change of a methoxy group to a hydroxyl group still enables the putative conformational changes and hydrogen bonding required for ribosome binding, this change results in increased hydrophilicity. Analyses of structure–activity relationships in this family of ribosome-binding compounds are providing evidence that greater toxicity is associated with increased hydrophobicity [35], which most likely accounts for the notable difference in the toxicities of diaphorin and pederin in mammalian, Sf9, and *S*. *cerevisiae* cells. It remains unclear why the toxicities of diaphorin and pederin are substantially different in these cells, but similar in *A*. *pisum* and *H*. *axyridis*. However, this discrepancy may provide clues regarding the more detailed mechanisms underlying the toxicities of these polyketides.

In *S*. *cerevisiae*, the ED_50_ of diaphorin and pederin was estimated as 252 ± 28 μM and 22.2 ± 2.2 μM, respectively (Fig 5, Table 2), indicating that *S*. *cerevisiae* is much less susceptible to these polyketides than mammalian cells. This is consistent with a previous report that indicated a treatment with approximately 10 μM pederin is required to inhibit *S*. *cerevisiae* cell growth, whereas nanomolar concentrations are sufficient to inhibit human cell growth [36]. This difference in the sensitivities of *S*. *cerevisiae* and human cells was assumed to be caused by variation in the permeability of target cells for pederin because cell-free protein-synthesizing systems derived from *S*. *cerevisiae* and mammalian cells reportedly exhibit approximately the same sensitivity [36]. This presumption may also be applicable to diaphorin, but further study is required to confirm this.

A single *D*. *citri* adult contains about 3 μg or approximately 6.5 nmol diaphorin (MW: 461.6) [13]. Because the average weight of *D*. *citri* adults is around 450 μg, their volumes can be approximated as 450 nL when assuming a specific gravity of 1.0. Thus, the diaphorin concentration within *D*. *citri* adults is estimated to be about 15 mM [13], which is two orders of magnitude higher than that required for toxicity to *S*. *cerevisiae*. Therefore, even though *S*. *cerevisiae* is relatively tolerant to diaphorin, the diaphorin concentration within *D*. *citri* should be sufficient to inhibit *S*. *cerevisiae* growth.

Among the organisms analyzed in this study, the lady beetle *H*. *axyridis* was the most resistant to diaphorin and pederin. Both diaphorin and pederin were toxic only at 5 mM (Fig 4), indicating that *H*. *axyridis* is three orders of magnitude more resistant to these polyketides than the pea aphid *A*. *pisum* (Fig 3). *A*. *pisum* is a phloem sap-sucking insect that is closely related to psyllids [23]. Additionally, the *A*. *pisum* genome has been affected by extensive gene duplications [23,37,38] as well as decreases in the number of defensive genes, including those related to the immune system and those encoding detoxification enzymes [23,39]. A relatively small set of genes related to detoxification may increase the susceptibility of *A*. *pisum* to toxins, although the relatively simple immune system of this aphid species may facilitate symbiotic relationships with microbes [23,25,40–47]. In contrast, *H*. *axyridis* is a major predator of *D*. *citri* [3,4], and a highly polyphagous carnivore [48]. This generalist lady beetle species encounters a diverse range of defensive chemicals from prey, and appears to be tolerant to these compounds [48–55]. The mechanism underlying the detoxification of these compounds remains largely uncharacterized, but it is likely to be a general process that is effective against the wide variety of toxins contained in various food sources [48]. Thus, it is reasonable that *H*. *axyridis* is highly resistant to diaphorin and pederin.

The average weight of the *H*. *axyridis* second instar larvae is about 2 mg, so their volumes can be approximated as 2 μL when assuming a specific gravity of 1.0. Thus, if a single *H*. *axyridis* second instar larva preys on a single *D*. *citri* adult, the maximum diaphorin concentration in the body of the predator will be about 3 mM (6.5 nmol/2 μL). This concentration is likely harmful for *H*. *axyridis*, as indicated by the results of the present study which demonstrated that 5 mM diaphorin is sufficient to kill this insect predator. The data presented herein were obtained following the injection of diaphorin into the *H*. *axyridis* body cavity. Consequently, there was no detoxification during digestion in the gut. However, a previous study revealed that five lady beetle species, including *H*. *axyridis*, exhibit a significantly poorer performance on a diet of *D*. *citri* than on a diet of *Ephestia kuehniella* (Lepidoptera: Pyralidae) eggs [56], implying diaphorin in *D*. *citri* ingested by feeding also has inhibitory effects on lady beetles.

The present study provides the new insights into the fact that the diaphorin, a polyketide synthesized by an intracellular symbiont of *D*. *citri*, is potentially harmful for biological control agents. It will be important to take this possibility into account in further investigations that aim to improve the efficacy of biological control of *D*. *citri*.
